# The Role of Perivascular Adipose Tissue in Early Changes in Arterial Function during High-Fat Diet and Its Combination with High-Fructose Intake in Rats

**DOI:** 10.3390/biomedicines9111552

**Published:** 2021-10-27

**Authors:** Jozef Torok, Anna Zemancikova, Zuzana Valaskova, Peter Balis

**Affiliations:** 1 Centre of Experimental Medicine, Institute of Normal and Pathological Physiology, Slovak Academy of Sciences, 813 71 Bratislava, Slovakia; zuzana.valaskova@savba.sk (Z.V.); peter.balis@savba.sk (P.B.); 2 Institute of Histology and Embryology, Faculty of Medicine, Comenius University, 811 04 Bratislava, Slovakia

**Keywords:** arteries, perivascular adipose tissue, high-fat diet, high-fructose intake, neurogenic contraction

## Abstract

The aim of the current study was to evaluate the influence of a high-fat diet and its combination with high-fructose intake on young normotensive rats, with focus on the modulatory effect of perivascular adipose tissue (PVAT) on the reactivity of isolated arteries. Six-week-old Wistar–Kyoto rats were treated for 8 weeks with a control diet (10% fat), a high-fat diet (HFD; 45% fat), or a combination of the HFD with a 10% solution of fructose. Contractile and relaxant responses of isolated rat arteries, with preserved and removed PVAT for selected vasoactive stimuli, were recorded isometrically by a force displacement transducer. The results demonstrated that, in young rats, eight weeks of the HFD might lead to body fat accumulation and early excitation of the cardiovascular sympathetic nervous system, as shown by increased heart rate and enhanced arterial contractile responses induced by endogenous noradrenaline released from perivascular sympathetic nerves. The addition of high-fructose intake deteriorated this state by impairment of arterial relaxation and resulted in mild elevation of systolic blood pressure; however, the increase in arterial neurogenic contractions was not detected. The diet-induced alterations in isolated arteries were observed only in the presence of PVAT, indicating that this structure is important in initiation of early vascular changes during the development of metabolic syndrome.

## 1. Introduction

Metabolic syndrome refers to the co-occurrence of several known cardiovascular risk factors, including central adiposity, hypertension, dyslipidemia, and impaired glucose metabolism. It has been associated with an increase in the incidence of coronary heart disease, stroke, and cardiovascular mortality [1,2]. With the rapid increase in the global prevalence of obesity, which is still in progress, the syndrome tends to affect large numbers of ever-younger people [3]. Moreover, the subjects who start to manifest metabolic syndrome in childhood, or in adolescence, will be exposed for decades to potent vascular risk factors, which suggests a high propensity for serious cardiovascular complications later in life [4,5].

To study the development of metabolic syndrome and its components, numerous animal models have been designed to date, using many genetic or environmental approaches [6,7]. Food regime and composition play an important role among the environmental factors, and various dietary schemes have been used to induce this syndrome in rodents, including a single type of diet or a combination of diets. It was suggested that the rat model displaying the closest criteria to human metabolic syndrome was induced by a high-carbohydrate, high-fat diet [6]. Among carbohydrates, fructose is of particular concern; it represents a simple ketonic sugar with slightly different mechanisms of absorption and metabolism compared to glucose, which are ultimately responsible for its highly lipogenic properties [8,9]. High-fructose feeding is often used as a model of insulin resistance, hyperinsulinemia, and hypertension, associated with the development of significant vascular impairments [10,11]. The mechanisms underlying a fructose-induced hypertensive state have not yet been fully characterized. Animal studies have shown that a high-fructose diet up-regulates sodium and chloride transporters, resulting in a state of salt overload; furthermore, it has also been found to activate vasoconstrictors, eliminate the effect of vasodilators, and over-stimulate the sympathetic nervous system (for review see [11]).

The long-term administration of higher doses of fructose is capable of inducing substantial cardiometabolic alterations in adult rats [12,13,14]; in contrast, younger individuals seem to be partially resistant to these changes, possibly due to more effective compensatory mechanisms [12]. However, the addition of high doses of fructose together with other types of diet in the early developmental phases of rats might predispose them to vascular stiffening and left ventricular diastolic dysfunction in later life [15].

Recent studies have highlighted the importance of perivascular fat in the development of vascular dysfunction and remodeling during the diet-induced increase in adiposity [16,17,18]. The perivascular adipose tissue (PVAT) is a functional component of the vasculature, exerting paracrine influences on vascular reactivity and proliferation. Under physiological conditions, the anticontractile effects of PVAT prevail in most arteries, attenuating vasoconstriction in rodents and humans through different mechanisms [19,20,21]. It was shown that in obese patients with metabolic syndrome, the total PVAT mass around small arteries was increased, while its anticontractile effect was completely lost, and markers of hypoxia and inflammation were detected [22]. Similar results were also obtained in animal genetic and diet-induced models of obesity [23,24,25]. Therefore, PVAT may represent one of the therapeutic targets for the development of vascular complications in obesity.

The aim of the present study is to determine the changes in reactivity of isolated arteries from normotensive Wistar–Kyoto rats after eight weeks of administration of a high-fat diet alone, or in combination with a high-fructose intake, starting at the sixth week of age to cover the adolescent period of development. The focus was put on the vasomodulatory effect of PVAT and its potential to contribute to the diet-induced alterations in arterial function.

## 2. Materials and Methods

### 2.1. Experimental Animals

The experiments were performed on young (6-week-old) males of the normotensive rat strain Wistar–Kyoto (WKY) (*n* = 18), housed at a temperature of 22–24 °C on a 12:12 h dark-light cycle (06.00–18.00 h light phase) with free access to laboratory rat chow and drinking water (ad libitum). The rats were divided into three experimental groups that were treated for the next 8 weeks in the following manner: a group fed with a control diet (*n* = 6; 10% energy from fat, formula C 1090-10, Altromin Spezialfutter, Lage, Germany), a group fed with a high-fat diet (HFD) (*n* = 6; 45% energy from fat, formula C 1090-45, Altromin Spezialfutter, Lage, Germany), and a combination of the HFD with a 10% solution of fructose replacing their drinking water (*n* = 6). The treatment period of 8 weeks was chosen based on some previous papers using high-fat or high-fructose diets [26,27,28] as well as on our precedent study that used high-fructose administration to adult rats where this period of treatment was effective to induce significant changes in arterial function [14].

Systolic blood pressure and heart rate were measured in conscious animals by the non-invasive tail-cuff method. The amount of food and fructose solution taken up by the rats was measured on a weekly basis to calculate the average daily energetic intake in the experimental groups. At the end of the treatment, rats were sacrificed by decapitation after brief CO_2_ anesthesia in a fasted state; before decapitation, their tail-tip blood was collected for measuring glucose concentration. The body weight, relative left heart ventricle, kidney, and liver weights, as well as the retroperitoneal and epididymal fat masses (normalized to tibia length), were determined in each rat. The thoracic and abdominal parts of the aorta and superior mesenteric artery were isolated from individual rats and prepared for isometric tension recording.

### 2.2. Functional Studies on Isolated Arteries

After removal from the rat, the aorta and superior mesenteric artery were quickly transferred to cold Krebs solution and cut into ring preparations (2.8–3.2 mm in length) of two types: one with preserved PVAT and the other with PVAT removed from the surface. In the case of rings with PVAT removed (PVAT(−)), the perivascular fat was removed under a microscope using fine scissors, being careful not to damage the adventitia. In the case of rings with PVAT preserved (PVAT(+)), a continuous layer of perivascular fat (1–1.2 mm thick) was left intact around the arterial ring. Special caution was taken not to damage the endothelial layer during preparation of each arterial ring.

The arterial rings were suspended in 20 mL organ baths filled with oxygenated (95% O_2_ + 5% CO_2_) modified Krebs solution maintained at 37 °C. The Krebs solution was prepared in the following composition (in mmol/L): NaCl 118, KCl 5, CaCl_2_ 2.5, MgSO_4_ 1.2, NaHCO_3_ 25, KH_2_PO_4_ 1.2, glucose 11, CaNa_2_ EDTA 0.03. The preparations were equilibrated under a resting tension of 10 mN for 60–90 min, and the Krebs solution was changed every 15 min.

Contractile and relaxant responses of arterial preparations to selected vasoactive or electrical stimuli were recorded isometrically by a Sanborn FT 10 force displacement transducer (Sanborn, Baltimore, MD, USA).

To assess arterial relaxation, cumulative concentration–response curves were obtained with an endothelium-dependent vasodilator acetylcholine (1 nM–10 μM) in rings of thoracic aorta precontracted with submaximal concentration of phenylephrine (1 μM).

Adrenergic contractions were determined in the abdominal aorta and mesenteric arteries as the response to exogenous noradrenaline (1 μM), or to endogenous noradrenaline released from periarterial sympathetic nerves (neurogenic contractions), by using transmural electrical stimulation (TES) or tyramine (0.1 mM), an indirectly acting sympathomimetic drug.

To perform electrical stimulation of periarterial sympathetic nerves, arterial rings were mounted between two platinum electrodes placed on either side of the preparation and connected to an electrostimulator ST-3 (Medicor, Budapest, Hungary). Frequency–response curves to electrical stimuli were obtained using square pulses of 0.5 ms in duration at supramaximal voltage (>30 V) applied at 1–32 Hz for a period of 20 s. The contractions of rat abdominal aortas and mesenteric arteries elicited by TES were blocked by guanethidine or tetrodotoxin, indicating that they were induced mainly by nerve-released (endogenous) noradrenaline.

The chemicals used in the protocol were purchased from Sigma-Aldrich (Darmstadt, Germany). All drugs were dissolved in distilled water, and their concentrations are expressed as the final concentration in the incubation chamber.

### 2.3. Data Analysis

The contractile responses were expressed as the active wall tension in mN and normalized to the length of the particular preparation (in mm). The dose-dependent relaxant responses to acetylcholine were expressed as a percentage of the phenylephrine-induced precontraction.

The values of area under curve (AUC, in arbitrary units) were calculated for individual concentration (frequency)–response curves (in mN) using the rectangular rule for numerical integration.

The results are presented as means ± standard errors of the means (SEM). Statistical evaluation was carried out by using one-way analysis of variance (ANOVA). The results were considered to be significant when *p* < 0.05.

## 3. Results

At six weeks old, before starting the treatment, the initial measurements in all rats (*n* = 18) showed the baseline values of body weight 119.9 ± 1.5 g, systolic blood pressure 101.6 ± 1.6 mm Hg, and heart rate 429.5 ± 9.7 bpm, with no differences among the three experimental groups.

There was no significant difference in the average daily food intake per rat during the whole treatment period between the control group and the group fed with the HFD (14.5 ± 0.4 g vs. 13.6 ± 0.4 g per rat, respectively; *p* > 0.05); however, the rat group receiving the HFD in combination with 10% fructose had significantly lower daily food consumption (10.5 ± 0.7 g per rat) compared to the group fed only with the HFD (*p* < 0.01). On the other hand, the average daily energetic intake in kilojoules was significantly higher in the HFD group compared to the control rats (256.4 ± 7.2 KJ vs. 213.2 ± 6.2 KJ per rat, respectively; *p* < 0.001), and significantly higher in the group treated with the HFD and 10% fructose together (299.2 ± 11.8 KJ per rat) compared to the group fed with the HFD alone (*p* < 0.01).

As presented in Table 1, eight weeks of the HFD alone, or in combination with 10% fructose, had no effect on the whole-body weight and on blood glucose in young Wistar–Kyoto rats. Similarly, it did not affect their relative heart and kidney weights.

In rats after treatment with the HFD alone, the retroperitoneal and epididymal fat pads were significantly enlarged compared to control rats. The HFD in combination with 10% fructose caused an increase in relative liver weight and in retroperitoneal fat weight compared to controls, indicating adipose as well as ectopic lipid accumulation. Both forms of treatment led to the elevation of heart rate in rats; however, systolic blood pressure was significantly increased only when the HFD was combined with 10% fructose (Table 1).

**Figure 1:**
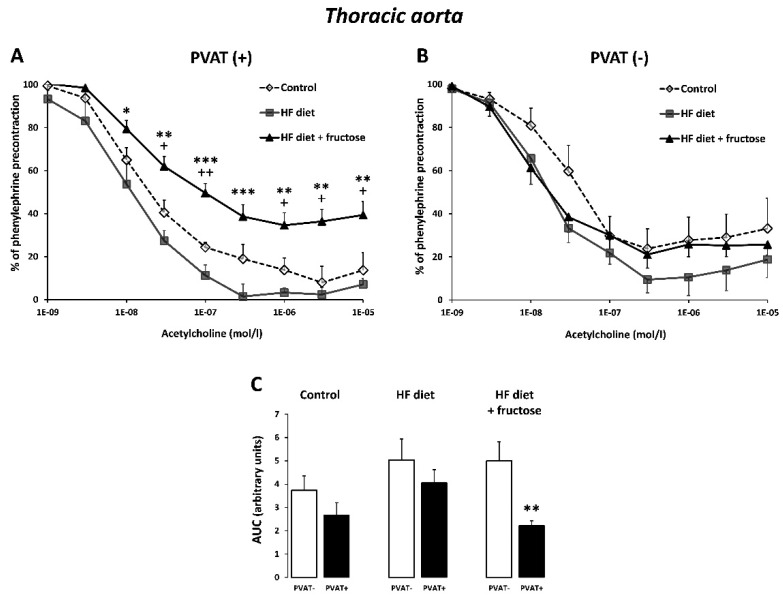
Endothelium-dependent relaxant responses to cumulative doses of acetylcholine in PVAT(+) (**A**) and PVAT(−) (**B**) preparations of thoracic aorta from Wistar–Kyoto rats: control, treated with the high-fat (HF) diet, and treated with the HF diet + fructose. * *p* < 0.05, ** *p* < 0.01, *** *p* < 0.001 vs. HF diet; + *p* < 0.05, ++ *p* < 0.01 vs. control. (**C**) Relaxations expressed as AUC (area under curve) values. ** *p* < 0.01 vs. PVAT(−). Values represent means ± SEM; *n* = 6.

In PVAT(−) preparations of rat thoracic aorta, the relaxant responses to acetylcholine were not significantly different among the three experimental groups (Figure 1B). In PVAT(+) rings, the acetylcholine relaxation was decreased in rats treated with the HFD in combination with fructose compared to other rat groups (Figure 1A). Only within this group receiving the combined treatment were the obtained AUC values of acetylcholine dose–response curves significantly lower in PVAT(+) compared to PVAT(−) preparations of thoracic aorta; in the control and the HFD-treated rats, the difference between PVAT(+) and PVAT(−) preparations was not significant (Figure 1C).

**Figure 2:**
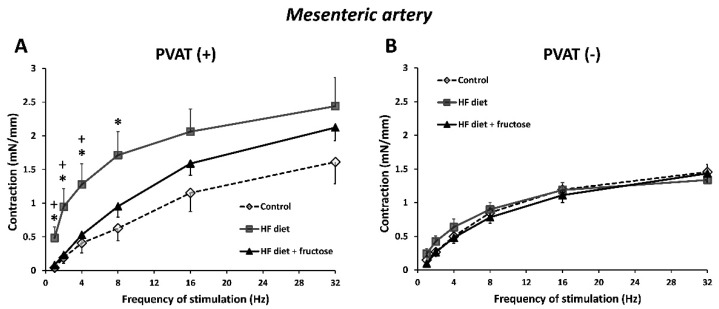
Frequency-dependent contractile responses to transmural electrical stimulation in PVAT(+) (**A**) and PVAT(−) (**B**) preparations of mesenteric artery from Wistar–Kyoto rats: control, treated with high-fat (HF) diet, and treated with HF diet + fructose. * *p* < 0.05 vs. HF diet + fructose; + *p* < 0.05 vs. control. Values represent means ± SEM; *n* = 6.

**Figure 3:**
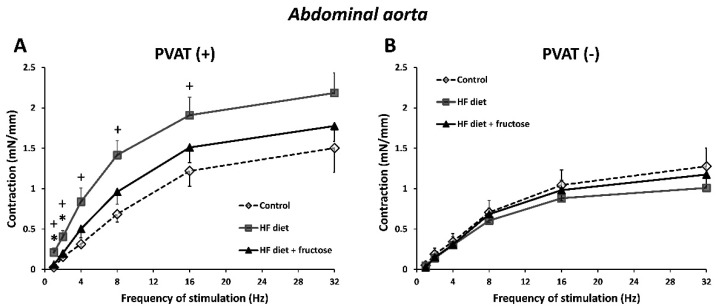
Frequency-dependent contractile responses to transmural electrical stimulation in PVAT(+) (**A**) and PVAT(−) (**B**) preparations of abdominal aorta from Wistar–Kyoto rats: control, treated with high-fat (HF) diet, and treated with HF diet + fructose. * *p* < 0.05 vs. HF diet + fructose; + *p* < 0.05 vs. control. Values represent means ± SEM; *n* = 6.

In both the mesenteric artery and abdominal aorta, the TES-induced neurogenic contractions were enhanced in PVAT(+) preparations in rats treated with the HFD alone when compared to other experimental groups (Figure 2A and Figure 3A). In PVAT(−) arterial rings, such differences were not detected (Figure 2B and Figure 3B).

**Figure 4:**
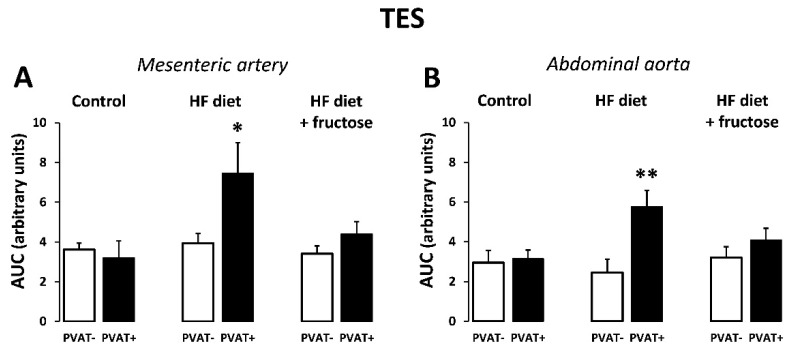
Frequency-dependent contractile responses to transmural electrical stimulation (TES) expressed as AUC (area under curve) values: comparison of PVAT(+) and PVAT(−) preparations of mesenteric artery (**A**) and abdominal aorta (**B**) in Wistar–Kyoto rats: control, treated with high-fat (HF) diet, and treated with HF diet + fructose. * *p* < 0.05, ** *p* < 0.01 vs. PVAT(−). Values represent means ± SEM; *n* = 6.

The AUC values obtained from frequency-dependent neurogenic contractions were higher in PVAT(+) compared to PVAT(−) preparations in the HFD-treated rats, though not in control or in fructose-combined HFD-treated rats (Figure 4).

**Figure 5:**
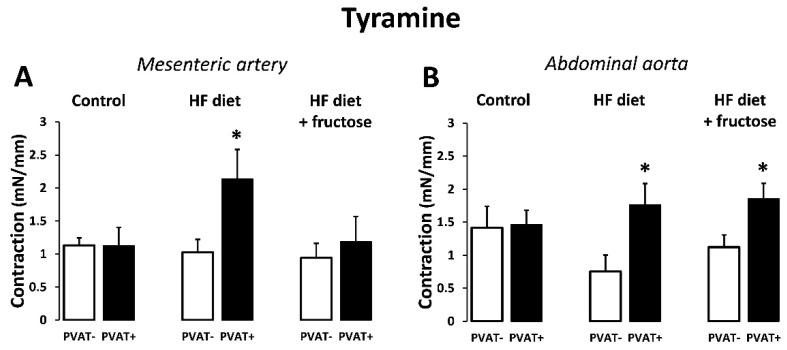
Contractile responses to tyramine (0.1 mM): comparison of PVAT(+) and PVAT(−) preparations of mesenteric artery (**A**) and abdominal aorta (**B**) in Wistar–Kyoto rats: control, treated with high-fat (HF) diet, and treated with HF diet + fructose. * *p* < 0.05 vs. PVAT(−). Values represent means ± SEM; *n* = 6.

Similarly, a single dose of the indirectly acting, sympathomimetic drug tyramine (0.1 mM) caused higher contractile responses in PVAT(+) compared to PVAT(−) preparations of mesenteric artery obtained from rats treated with the HFD alone (Figure 5A), as well as in preparations of abdominal aorta from rats treated with the HFD alone or in combination with 10% fructose (Figure 5B).

**Figure 6:**
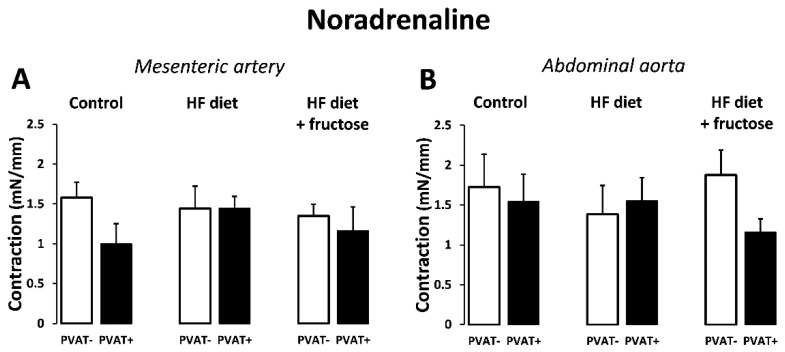
Contractile responses to exogenous noradrenaline (1 μM): comparison of PVAT(+) and PVAT(−) preparations of mesenteric artery (**A**) and abdominal aorta (**B**) in Wistar–Kyoto rats: control, treated with high-fat (HF) diet, and treated with HF diet + fructose. Values represent means ± SEM; *n* = 6.

The contractile responses of mesenteric arteries and abdominal aortas to a single dose of exogenous noradrenaline (1 μM) were not different among the experimental rat groups; the differences were not significant even between PVAT(+) and PVAT(−) arterial preparations within the particular groups (Figure 6).

## 4. Discussion

In this study, we documented that eight weeks of a high-fat diet and its combination with high-fructose treatment in young Wistar–Kyoto rats led to increased lipid storage in adipose tissue and to ectopic lipid accumulation in the liver, as well as to some early changes in the reactivity of isolated arteries. However, the treatment did not alter the level of glycaemia, indicating that the metabolic syndrome was only in the initial phase of its development. The increase in body adiposity and the incipient cardiovascular impairment detected in this experiment using adolescent rats were comparable to the HFD- and high-sugar diet-induced alterations in other adult animals [16,29]. According to the theory of developmental windows and epigenetic programming in early postnatal phases, however, one can assume that diet-induced changes at a young age could considerably influence metabolic processes and vascular functions in adulthood [15,30,31].

The differences in arterial reactivity observed in this study after the HFD or combined-fructose treatment were specifically associated with the presence of intact perivascular fat, while the arteries cleaned from PVAT showed similar functional properties when compared to PVAT(−) arterial preparations obtained from control rats. This might indicate that the treatment did not affect the arteries themselves, but that the diet-induced alterations might influence the direct paracrine effect of PVAT on arterial responses. It can be presumed that the prolongation of the treatment could lead to further significant changes in the structure and function of the cardiovascular system due to the developing diabetic state, as shown by Lozano et al. [32].

Similar to the present results, our previous studies demonstrated that long-term, high-fructose intake in rats led to the growth of body fat content, liver weight, and to a moderate rise in blood pressure, indicating that fructose alone might induce such alterations progressively leading to metabolic syndrome [14,33]. Moreover, the observed changes due to fructose were accompanied by the considerable attenuation of sympathoadrenergic activity in isolated arteries with intact PVAT [14], which is in contrast with the augmentation of neurogenic contractions by the HFD seen in this study. As shown in the present results, the addition of 10% fructose to the HFD caused elimination of the enhancement of sympathetic contractions observed in the HFD diet alone, confirming again the attenuating effect of high-fructose treatment on arterial sympathoadrenergic responses.

Although both an HFD and high-fructose intake induce similar metabolic–obesogenic changes resembling the initiation of metabolic syndrome, their synergistic or cumulative effect in adolescent rats fed with the HFD and drinking 10% fructose was not confirmed. One of the reasons could be the lower amount of high-fat food taken up by the rats treated with the HFD and fructose solution together when compared with the group fed only with the HFD, which might be considered as a limitation of this study. On the other hand, the opposite effects of these two types of diet on arterial sympathetic responses indicate that their mechanisms of action might be different in some regards. For example, Huang et al. [34] found out in their study that high-fat food resulted in an impaired pancreatic insulin secretion function; in contrast, a high-fructose diet caused hyperinsulinemia and insulin-stimulated high secretion of leptin. Increased levels of leptin produced by the expanded body fat were observed in obese humans as well as in studies with an obesogenic diet applied to animals [35,36]; it is supposed that this substance might represent a possible link between high fat accumulation and an increase in sympathoneural activity, leading to an obesity-induced hypertensive state [37,38]. In the present study, the higher level of sympathetic excitation in rats treated with the HFD was indicated by the increased heart rate and the enhancement of sympathoadrenergic contractions in PVAT(+) arterial preparations. In contrast, the additional high-fructose treatment resulted in attenuation of arterial sympathetic contractions; however, a mild elevation of heart rate and systolic blood pressure was detected in these rats.

The diet-induced differences in adrenergic contractions were observed only in the presence of intact PVAT during the release of endogenous noradrenaline from the perivascular sympathetic nerves (electrically or tyramine-induced release). In contrast, no significant changes were detected when comparing the contractions to exogenous noradrenaline in preparations with intact or removed PVAT. This might confirm that the sensitivity of isolated arteries to adrenergic stimuli was not altered and that the detected changes were caused by the increased release of noradrenaline from endogenous neural sources in PVAT. The enhanced responses to tyramine, an indirectly acting sympathomimetic drug, in the presence of PVAT again support this concept.

From the abovementioned results it seems that during the development of metabolic syndrome and associated cardiovascular impairment, the alterations in arterial function could be initiated within the expanding PVAT and later be progressively manifested in the proper arterial wall. To support this presumption, in our recent studies, we have revealed that in older Zucker diabetic fatty (ZDF) rats with developed severe metabolic syndrome, their arteries cleaned from PVAT showed significantly increased sensitivity in contractile responses to adrenergic stimulation [39] when compared to control non-obese rat groups. Interestingly, in ZDF rats’ mesenteric arteries, the perivascular fat exerts rather anticontractile properties and possibly compensates for the long-term procontractile state in arteries as a result of increased sympathetic tone and impaired dilatation due to extensive cardiometabolic disease.

Similarly, the presented observations confirmed the reduced endothelium-dependent relaxation of thoracic aorta in rats receiving the HFD combined with fructose, but only in aortal preparations with preserved PVAT. In contrast, the isolated thoracic aortas from old ZDF rats showed damaged acetylcholine-induced relaxation in PVAT(−) preparations [40], clearly displaying the impairment within the arterial wall after the long-term influence of metabolic syndrome.

Arterial endothelial dysfunction was described in studies using different fructose-fed protocols, and it was related to increased oxidative stress, reduced production or availability of nitric oxide, and increased levels of uric acid and endothelin-1 [41,42,43]. The presented results as well as the observations of other authors demonstrate that the substances released from PVAT could participate in these pathological processes [17,44].

Less explained is the inhibitory effect of an eight-week, high-fructose diet on adrenergic contractions, seen in our preceding work [14], and being indirectly confirmed in the present study by attenuating the enhancement of neurogenic contractions caused by the HFD. Previously, Abdulla et al. [45] suggested that the long-term sympatho-excitation due to chronic high-fructose intake blunts the responses of the renal vasculature to adrenergic agonists. According to the authors, this reduction appears to result from a decrease in adrenergic receptor sensitivity as well as a reduced responsiveness to angiotensin II, being suggestive of a defect in intracellular signaling.

In the present study, however, the attenuation of neurogenic responses in rats receiving fructose was observed in PVAT(+) arterial preparations, indicating that the perivascular fat could be the source of the potential vasorelaxant substance(s) decreasing adrenergic contractions. Besides a number of PVAT-derived anticontractile molecules (e.g., adipokines, cytokines/chemokines, gaseous molecules, angiotensin 1–7, methyl palmitate, and reactive oxygen species) (for review, see [17]), which are able to regulate the developed contractions predominantly in a physiological state, there might even be other structures, possibly of neural origin, which can counteract the sympathetic contractions during electrical nerve stimulation. It was documented that, except for the perivascular sympathoadrenergic nerves, the activity of which can be specifically blocked with guanethidine, the presence of sensory nerves containing calcitonin gene-related peptide (CGRP) and other neurotransmitters was also confirmed in the surface layers throughout the vascular system, especially in the mesenteric arterial bed. During TES, they are responsible for the pronounced neurogenic vasorelaxation in mesenteric and other types of arteries [46,47,48]. Several papers brought evidences that PVAT can be innervated by both sympathetic [49,50,51,52] and sensory nerves [47], the activity of which is differently manifested in various metabolic situations and vessel types and is importantly influenced by the substances produced by the PVAT adipocytes. It might be presumed that during electrical stimulation of perivascular nerves in isolated arterial preparations, the sensory nerve activity inhibits the resultant sympathetic contraction, an effect which could be responsible for the elimination of the enhancement of arterial contractile responses to TES when the HFD is combined with high-fructose intake.

## 5. Conclusions

The presented results demonstrate that in young rats, the eight-week, high-fat diet might lead to body fat accumulation and early excitation of the cardiovascular sympathetic nervous system. These changes could further lead to the development of metabolic syndrome in adulthood. The addition of high-fructose treatment caused the impairment of arterial relaxation and resulted in a modest elevation of systolic blood pressure; however, it eliminated the increase in arterial sympathetic contractions which was observed after the treatment with the high-fat diet alone. The diet-induced alterations in isolated arteries were observed only in the presence of intact perivascular adipose tissue, indicating that this structure is important in the initiation of early vascular changes during the development of metabolic syndrome.

## Figures and Tables

**Table 1:** Selected cardiovascular and biometric characteristics of 14-week-old Wistar–Kyoto rats: control, treated with high-fat (HF) diet, or treated with HF diet in combination with 10% fructose.

	Control	HF Diet	HF Diet + Fructose
Body weight (g)	273.2 ± 9.1	301.7 ± 13.8	279.6 ± 12.2
Systolic blood pressure (mm Hg)	114.8 ± 2.6	120.1 ± 1.7	123.7 ± 2.7 *
Heart rate (bpm)	412.3 ± 5.0	440.5 ± 8.1 *	439.5 ± 8.8 *
Heart weight/tibia length (mg/mm)	28.2 ± 1.2	30.2 ± 1.0	29.1 ± 0.6
Kidney weight/tibia length (mg/mm)	31.1 ± 0.6	31.1 ± 1.2	30.2 ± 0.4
Liver weight/tibia length (mg/mm)	184.8 ± 5.7	192.9 ± 7.4	202.6 ± 3.3 *
RP fat weight/tibia length (mg/mm)	34.4 ± 7.6	91.5 ± 13.2 *	63.6 ± 4.0 *
ED fat weight/tibia length (mg/mm)	46.7 ± 4.5	75.1 ± 8.1 *	54.2 ± 1.9
Glucose (mmol/L)	5.2 ± 0.2	5.4 ± 0.2	5.1 ± 0.1

bpm—beats per minute; RP fat—retroperitoneal fat; ED fat—epididymal fat. Values represent mean ± SEM; *n* = 6; * *p* < 0.05 vs. control.

## Data Availability

The data presented in this study are available in this manuscript.
